# Biomarkers in Oral Fluids as Diagnostic Tool for Psoriasis

**DOI:** 10.3390/life12040501

**Published:** 2022-03-29

**Authors:** Constanza Jiménez, María José Bordagaray, José Luis Villarroel, Tania Flores, Dafna Benadof, Alejandra Fernández, Fernando Valenzuela

**Affiliations:** 1 Faculty of Dentistry, Universidad Andres Bello, Santiago 8370133, Chile; c.jimenezlizama@uandresbello.edu (C.J.); dafna.benadof@unab.cl (D.B.); 2 Department of Conservative Dentistry, Faculty of Dentistry, Universidad de Chile, Santiago 8380544, Chile; mbordagaray@odontologia.uchile.cl; 3 Department of Dermatology, Faculty of Medicine, Universidad de Chile, Santiago 8380453, Chile; jose.villarroel@ug.uchile.cl; 4 Research Centre in Dental Science (CICO), Faculty of Dentistry, Universidad de La Frontera, Temuco 4780000, Chile; tania.flores@ufrontera.cl

**Keywords:** psoriasis, biomarkers, saliva, gingival crevicular fluid

## Abstract

Psoriasis is a prevalent worldwide chronic immuno-inflammatory skin disease with various variants and atypical cases. The use of biomarkers for the diagnosis of psoriasis can favor timely treatment and thus improve the quality of life of those affected. In general, the search for biomarkers in oral fluids is recommended as it is a non-invasive and fast technique. This narrative review aimed to identify biomarkers in gingival crevicular fluid (GCF) and saliva to diagnose psoriasis. To achieve this goal, we selected the available literature using the following MESH terms: “psoriasis”, “saliva” and “gingival crevicular fluid”. The studies analyzed for this review cover original research articles available in English. We found three full articles available for psoriasis biomarkers in GCF and ten articles available for psoriasis biomarkers in saliva. Studies showed that in the saliva of healthy individuals and those with psoriasis, there were differences in the levels of inflammatory cytokines, immunoglobulin A, and antioxidant biomarkers. In GCF, individuals with psoriasis showed higher levels of S100A8, IL-18 and sE-selectin in comparison to healthy individuals, independent of periodontal status. Despite these findings, more studies are required to determine an adequate panel of biomarkers to use in saliva or GCF for psoriasis.

## 1. Introduction

Psoriasis is a chronic immuno-inflammatory skin disease that, if symptomatic and untreated, can diminish the quality of life of affected individuals [1,2]. The prevalence of psoriasis is high and varies between 0.63% to 3.60% in North America, 0.36% to 2.96% in Southern Latin America, and 0.62% to 5.32% in Central Europe [3]. Moderate and severe psoriasis are more frequent in men than women [4]. Its etiopathogenesis is still unknown; however, studies have shown its association with the presence of the polymorphism of inflammatory genes and alterations in the skin microbiome, which can deregulate the immune system response [5,6].

Psoriasis is characterized by the proliferation of keratinocytes leading to squamous plaques [7]. Nevertheless, ocular, nail, and even oral manifestations may be involved [8,9,10]. Moreover, psoriasis can cause low-grade systemic inflammation with increased levels of inflammatory cytokines and C-reactive protein levels (CRP) [11,12]. Emerging evidence also shows a higher cardiovascular risk in individuals with this disease [13].

CD4 T cells have an essential role in the pathogenesis of psoriasis, even more than CD8 T cells, being present in a higher proportion in compromised skin [14]. One study showed that mice transplanted with activated helper lymphocytes from patients with psoriasis later developed psoriatic plaques [15]. Other studies have shown a higher production of cytokines with a T-helper 1 (Th1) profile, such as interferon (INF)-γ, tumor necrosis factor (TNF)-α, and interleukin (IL)-2, without a significant elevation in the T-helper 2 (Th2) profile [14,16]. However, its pathogenesis is not wholly explained by the Th1 route, and therefore new research has been set to further study this issue. For example, investigations have shown that Th17 cells produce IL-17 and TNF-α [17], and are activated mainly by IL-6 and tumor growth factor (TGF)-β [18]. Furthermore, the action of IL-23, produced by dendritic cells and monocyte/macrophages [19,20], perpetuates the activity of Th17 cells, with the subsequent secretion of IL-17 and TNF-α. This generates hyperproliferation of keratinocytes and higher recruitment of immune cells, and magnifying the inflammatory process [21]. 

Psoriasis diagnosis is primarily clinical, based on skin lesions such as pruritic, scaly erythematous plaques. The severity of this disease is determined by the extent of body surface affected, erythema, induration, and scaling [22]. Differential diagnoses include atopic dermatitis, contact dermatitis, lichen planus, secondary syphilis, mycosis fungoides, tinea corporis, and pityriasis rosea [22,23]. Health professionals should consider a skin biopsy for differential diagnosis of psoriasis; however, it is an invasive process and does not provide information about the stage of the disease [24]. Therefore, molecules in the skin and body fluids have been investigated to support the diagnosis of psoriasis [25,26,27]. Among the different body fluids that allow the detection of psoriasis biomarkers, the use of GCF and saliva stands out for being non-invasive and rapid methods [25,27]. This review identifies biomarkers in GCF and saliva to diagnose psoriasis as described in the literature.

## 2. Search Strategy

**Figure 1:**
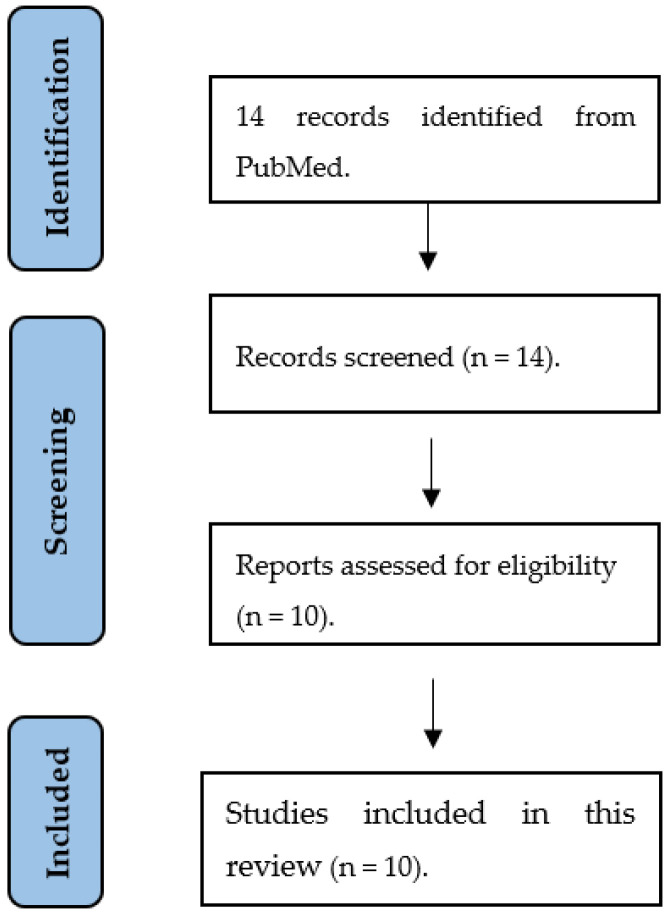
Flowchart describing study selection for salivary biomarkers as a diagnostic tool for psoriasis.

We found 17 articles in the electronic database PubMed, using the following MESH terms: (1) “psoriasis” AND “gingival crevicular fluid”; (2) “psoriasis” AND “saliva”. The inclusion criteria were original research articles fully available in English. The exclusion criteria were letters to the editor, case reports, in vitro studies, and clinical trials. We found three full-text articles available for psoriasis biomarkers in GCF, which were selected. The search strategy for psoriasis biomarkers in saliva is described in Figure 1; ten of the 14 screened articles were included.

## 3. Gingival Crevicular Fluid Biomarkers as a Diagnostic Tool for Psoriasis

Biomarkers are molecules that may be collected from different biological sources. They are used to evaluate physiological and pathological conditions, as well as clinical and pharmacological responses to therapeutic interventions [28]. Different concentrations or alterations in the production or function of proteins, lipids, DNA/RNA, serve as biomarkers for diagnosing and prognosing several illnesses in the biomedical fields [29,30]. In addition, biomarker screening may be used to identify an individual’s susceptibility to a particular disease [31]. With recent advances in proteomics and molecular biology, oral fluids have been recognized as novel, non-invasive, and readily available biomarker sources for oral and systemic disease diagnosis [32,33].

GCF is a serum transudate produced and secreted into the gingival crevice between the tooth surface and the epithelial integument [34]. Its primary function is to cleanse foreign materials and microorganisms from the gingival-dental junction, acting as a natural barrier against bacterial invasion [34]. The production of GCF achieves this at a tiny yet constant rate, known as GCF flow. Two critical aspects of GCF, as a biomarker source, are its rather unique composition and relative isolation from the oral environment. The GCF flow prevents outside substances from penetrating the gingival sulcus [35,36], while promptly washing and expelling foreign materials that do enter [37,38]. Retrograde salivary flow into the gingival crevice is also rare, as GCF flow inhibits the entrance of salivary contents into the gingival-dental sulcus. In addition, several studies have shown that GCF samples (>70%) usually lack salivary amylase [39], and immunoglobulin (Ig) G concentration can reach almost 100 times that of saliva [40]. These results could not be possible if saliva gained easy access to the gingival sulcus.

Under healthy conditions, GCF consists of a mix of serum and locally produced proteins. Its production is scarce and primarily interstitial because of the osmotic gradient that flows from the vascularized connective tissues to the gingival epithelium and crevice [41]. Locally produced molecules found in the GCF include microbial by products of the subgingival biofilm (i.e., endotoxins) and soluble molecules such as antibodies, enzymes, and organic/inorganic ions produced by periodontal cells, leukocytes, and physiological tissue breakdown. These elements give an essential insight into periodontal metabolism and the adaptive responses of subgingival bacteria within the gingival crevice [32].

Under pathological conditions, however, the volume and composition of the GCF change into a profuse inflammatory exudate [42,43]. GCF exudate accompanies periodontal inflammation and precedes its hallmark clinical signs [44]. In addition, its immediate vicinity to the periodontal tissues, site-specificity, and readily available access makes it the ideal biomarker source for early diagnosis and prognosis of periodontal diseases. To this date, over 90 different biomarkers with clinical and therapeutic value have been successfully identified in the GCF [45], most of which are cytokines and enzymes produced by periodontal tissue destruction and inflammatory active polymorphonuclear neutrophils and lymphocytes [46]. Some of these cell products have been associated with periodontal disease severity [42,43], whereas others show a positive correlation with clinical parameters before and after periodontal treatment, suggesting a valuable use for the assessment of periodontal treatment outcomes [47].

Over the last decade, the advent of “precision medicine” has focused on using biological profiles (i.e., genomic, proteomic, transcriptomic, among others) to personalize the diagnosis and treatment of systemic diseases. Recently, particular interest has fallen in the profiling of GCF in health and disease. Previous studies using ELISA and multiplex-bead immunoassay techniques have reported the existence of different “GCF profiles” between systemically healthy subjects and individuals with diabetes, rheumatoid arthritis, acute myocardial infarction, and end-stage renal disease [48,49,50,51,52]. Interestingly, some of these differences were found regardless of periodontal status [48,51,52]. Our research group has experience exploring the GCF of patients with several dermatoses [53]. In a previous study focused on patients with moderate/severe atopic dermatitis (AD), we found significantly lower MMP8 levels in GCF of AD patients versus healthy controls. The area under the receiver operating characteristic (ROC) curve for MMP8 was 0.672, *p* < 0.05 [53].

Studies exploring the GCF of psoriatic patients are still scarce (for available articles see Table 1). In an article published in 2013, researchers explored the impact of autoimmune diseases (particularly rheumatoid arthritis, psoriasis, and systemic sclerosis) and anti-TNF-α therapy on the clinical and immunological periodontal parameters of diseased subjects and systemically healthy controls [54]. Regarding psoriasis exclusively, researchers found that the levels of GCF TNF-α were significantly higher in psoriatic patients compared to healthy controls. Nevertheless, since probing depth and gingival index periodontal parameters were also substantially worse in the psoriasis group (>0.002), it is difficult to establish whether the over-expression of crevicular TNF-α reflects the systemic disease or the local inflammatory response of the periodontium [54].

A second study conducted by our research group in 2021 explored the GCF levels of IL-17A, IL-22, IL-23, and the S100 proteins A7, A8, and A9 in psoriatic subjects and systemically healthy controls with and without periodontitis [29]. Within this study, psoriatic patients presented significantly higher GCF levels of S100A8 than systemically healthy controls, regardless of periodontal status/health. Furthermore, a positive correlation was observed between crevicular S100A8 concentrations and psoriasis severity, indicating that S100A8 is not only a central protein of psoriasis pathogenesis but also a plausible biomarker for future diagnostic and therapeutic strategies for the assessment of the dermatosis. No significant intergroup differences in the crevicular expression of Il-17A, IL-22, IL-23, and S100A7 were noticed, whereas S100A9 concentrations exceeded the detection limits of the immunoassay in all groups [29].

Finally, a third study further expanded on the characterization of GCF in psoriatic patients. Moderate/severe psoriatic patients presented significantly higher and lower concentrations of IL-18 and soluble E-selectin in comparison to healthy control, respectively. In contrast, no intergroup differences were noticed in the soluble ICAM-1 crevicular level. Interestingly, periodontal status did not affect the GCF concentrations of IL-18 and soluble E-selectin, as seen by using a multiple regression statistical model. The ROC curve for IL-18 showed a 0.77 area with a sensitivity and specificity of 73.81% and 64.10% each. On the other hand, soluble E-selectin presented a ROC area of 0.68 with a sensitivity and specificity of 90.48% and 61.54%, respectively. Overall, our results regarding the ROC curve area of IL-18 in GCF were similar to those previously reported for serum samples of psoriatic patients [55]; hence, changes in the expression of the protein might be reflecting the systemic inflammatory burden of the dermatosis.

Ultimately, it is essential to acknowledge that all articles presented in this manuscript are cross-sectional studies. As such, they do not allow for causality associations. In addition, psoriasis is a complex disease possibly triggered by genetic, epigenetic, and proteomic imbalances, as well as by infectious and microbiological factors. Likewise, cytokines, enzymes, and other biological products rarely exert their biological functions as single molecules; hence, the prospective biomarkers identified in this review are more helpful to analyze (GCF profiling) than by separate. Because most biological markers identified in this review are also universal biomarkers of inflammation, results must be interpreted with caution since changes in their concentrations might be reflecting other systemic and/or local inflammatory conditions aside from psoriasis. Finally, multiplex-bead immunoassays could be a helpful laboratory technique to achieve what has been proposed. It allows for simultaneously checking the presence and quantity of several proteins within a single sample [56].

## 4. Salivary Biomarkers as a Diagnostic Tool for Psoriasis

Saliva is a clear fluid constituted mainly for water followed by proteins and some inorganic substances created by major and minor salivary glands [57,58]. Due to its composition, it is used to search for biomarkers of oral and systemic diseases [27,59]. The advantage of searching for biomarkers in saliva compared to blood is that it is a fast and non-invasive method to diagnose the onset and progression of several systemic diseases [60,61,62]. It is possible that the biomarkers circulating in the blood enter the saliva through the permeable capillaries present in the salivary glands and then, all together, are released into the oral cavity [63]. Accordingly, the study of salivary biomarkers might demonstrate a global picture of the body [60].

Studies performed in psoriatic individuals have shown a differential molecular, immunological, and microbial composition and amount of saliva secretion compared to healthy controls. These studies, summarized in Table 2, possibly contribute to understanding the oral physiological consequences of psoriatic individuals. In this matter, the levels of TNF-α, IL-12, and IFN-γ in the salivary milieu in psoriatic individuals are elevated while the levels of IL-10 are reduced compared to healthy controls [64]. This cytokine profile favors the hypothesis of an imbalance between Th1 and Th2 cells in the salivary glands of psoriasis individuals, previously reported in psoriatic skin lesions [65]. The salivary levels of IL-1β are also higher in psoriatic individuals, remaining unaltered in stressful situations [66], but being reduced after TNF-α inhibition medication compared with the same individuals before the treatment [27]. In addition, the IL-1β levels in the saliva of psoriatic patients positively correlate with psoriasis activity [27]. Accordingly, the measurement of IL-1β levels in saliva could be helpful as a non-invasive tracking method of the progression of the disease in psoriatic individuals.

Also, the secretory function of the salivary glands is diminished in psoriatic individuals compared to matched sex and gender healthy controls [64]. In consideration of the secretory function, ROC analysis of the nitrosative stress markers has demonstrated that nitric oxide (0.77), nitrotyrosine (0.74), and IL-2 (0.81) levels in saliva could differentiate hypo versus normal salivary flow in psoriatic individuals [64]. Concerning dimensional alterations, infrared spectrophotometry analysis has demonstrated that the secondary structure of compositional salivary proteins in plaque psoriasis individuals is altered compared to healthy counterparts [67]. The protein dimensional structure changes in plaque psoriasis saliva are similar to diabetes individuals [67], enhancing the evidence linking plaque psoriasis as a multi-systemic disorder related to diabetes.

The immunological features of the saliva of psoriasis individuals have also been studied. Controversial evidence has shown higher [68] and lower [69] IgA levels in saliva of psoriatic subjects in comparison with controls, while the levels of IgM and IgE are similar in both groups of study [68]. On the other hand, evidence supports lower levels of lysozyme in the saliva of psoriatic individuals compared to controls [69,70]. Overall, the changes in the immunological composition of saliva in psoriasis could contribute to the establishment of differential microbial communities, as has been recently published and discussed in detail below.

In terms of microbiological composition, the saliva of psoriatic individuals has demonstrated a differential salivary microbiota. The 16s ribosomal RNA sequencing technique and posterior linear discriminant analysis have demonstrated that 13 and 8 taxa are associated with healthy and psoriatic individuals, respectively [71]. The taxa *Actinomyces*, *Saccharibacteria*, *Streptococcus*, and *Lactobacillus* are associated with healthy individuals, while *Prevotella, Neisseria*, and *Agreggatibacter* are associated with the salivary microbiota of psoriatic subjects [71].

**Table 2:** Biomarkers of psoriasis in saliva.

Autor, Year	Study Design	Population	Comparison (Control)	Technique	Biomarker Level	*p*
Skutnik-Radziszewska et al., 2020 [64]	Cross-sectional	Blood and saliva samples from 60 patients with psoriasis were divided into two groups: patients with psoriasis and hyposalivation (*n* = 30) and patients with psoriasis and normal secretion (*n* = 30).	Healthy controls (*n* = 60)	Biochemical assays, DNS method, BCA method, spectrophotometry, ELISA	Elevated levels of TNF-α, IL-2, and INF-γ, and reduced levels of IL-10 in psoriasis compared to the control group.	**<0.05**
Belstrøm, D. et al., 2020 [71]	Cross-sectional	Stimulated saliva samples from patients with psoriasis (*n* = 27), patients with periodontitis (*n* = 58).	Healthy controls (*n* = 52).	Immunoassays, rRNA sequence analysis.	Linear discriminant effect size analysis showed that 52 bacterial taxa (22 psoriasis and 30 periodontitis) and 21 bacteria taxa associated with the healthy control differentiated the salivary microbiota of patients with psoriasis from that of orally healthy patients with periodontitis. Significantly lower mean salivary levels of NGAL (psoriasis: 996, periodontitis: 2072, controls: 2551 ng/mL) and transferrin (psoriasis: 4.37, periodontitis: 7.25, controls: 10.02 ng/mL).	**<0.0001**
Ganzetti, G. et al., 2016 [27]	Prospective with follow-up	Saliva samples from patients with psoriasis (*n* = 25) and control subjects (*n* = 20).		Βiochemical assays for detection of interleukin IL-1 *β* levels.	IL-1*β* levels in saliva of patients with psoriasis were significantly higher than in healthy controls. In patients with psoriasis, TNF-α inhibitor treatment significantly reduced IL-1*β* levels, compared with baseline. There is a positive correlation between IL-1*β* levels and psoriasis activity.	**<0.05**
Bottoni, U., et al., 2016 [67]	Cross-sectional	Saliva samples from 35 patients with psoriasis, 20 patients with diabetes.	Healthy subjects (*n* = 20).	Infrared spectrometry.	Presence of a structural alteration of proteins in the saliva of patients with psoriasis similar to that observed in patients with diabetes. This suggests that psoriasis is a multisystemic disorder strictly related to diabetes.	**<0.05**
Mastrolonardo, M., et al., 2007 [66]	Cross-sectional	Saliva samples from patients with psoriasis (*n* = 25) of mild to moderate severity.	Healthy subjects (*n* = 50).	HS salivary cortisol enzyme immunoassay, ELISA.	IL-1*β* levels were significantly higher in psoriasis individuals than controls, while cortisol levels did not differ significantly between groups. There was no significant correlation between changes in IL-1*β* and cortisol levels in psoriasis patients or controls.	**<0.05**
del Castillo, L.F. , et al., 1981 [68]	Prospective with follow-up	Blood (right cubital vein) and saliva (right parotid gland) samples from psoriasis patients (*n* = 10).	Healthy volunteers (*n* = 10).	Radioimmunological analysis PRIST, laser nephelometry.	IgA levels were significantly higher in psoriatic patients than controls. The mean IgG and IgM levels determined in both groups did not differ significantly from each other.	**<0.01**
Syrjainen, S.M. 1983 [70]	Cross-sectional	Lacrimal and salivary fluid samples from psoriatic patients (*n* = 28).	Healthy controls (*n* = 10).	Photometric and colorimetric analysis, Phadebas method, radial immunodiffusion analysis and radioimmunoassay.	Increased levels of amylase, Na+ and IgA in patients with psoriasis, as well as reduced lysozyme levels.	**<0.001**
Skutnik-Radziszewska et al., 2020 [64]	Cross-sectional	Blood and saliva samples from patients with psoriasis (*n* = 40).	Healthy controls (*n* = 40).	Biochemical assays, dental examination, redox analysis, BCA method, colorimetric analysis, chemiluminescence test.	The antioxidant enzyme activity, protein oxidation markers concentration, and reactive oxygen species production rate in psoriasis patients were significantly higher than in controls.	**<0.001**
Soudan, R.A. et al., 2011 [72]	Cross-sectional	Saliva samples from patients with psoriasis (*n* = 20).	Healthy controls (*n* = 20).	Biochemical assays.	Significantly higher concentrations of K+ and sAA (mean K+ = 21.38 mmol/L, mean sAA = 64.26 IU/mL) in patients than in controls.	**<0.05**
Koh, D. et al., 2004 [69]	Cross-sectional	Saliva samples from patients with psoriasis (*n* = 51) and control subjects (*n* = 24).	Healthy controls (*n* = 24).	Enzyme-linked immunosorbent assay.	Psoriasis patients had a lower concentration and secretion rate of IgA and lysozyme than controls.	**0.000**

IL, Interleukin. Bold, significant *p*-values.

## 5. Conclusions

Psoriasis is a common worldwide disease that is mainly diagnosed clinically. However, its manifestations can be confused with other conditions, leading to erroneous treatment. An excellent alternative to support clinicians in determining the diagnosis and severity of diseases is to measure the concentration of biomarkers in body fluids. In this sense, the search for psoriasis biomarkers in saliva and GCF has increased. The search for psoriasis biomarkers in oral fluids has been more common in saliva than GCF. Both alternatives are quick and painless; however, the search for biomarkers in the GCF requires taking a sample by a trained professional and a periodontal evaluation.

Oral biofluids have several advantages over conventional skin biopsy biomarker screening: First, it is a non-invasive method with an easy, quick, and less time-consuming collection that may be performed in basic clinical settings. Second, saliva and gingival crevicular fluid samples are much easier to handle and store due to their small volume, which significantly reduces the risk of operator contamination. Third, oral biofluid samples unveil many biomarkers (including those derived from plasma ultrafiltrate) and thus may reflect several systemic and oral conditions simultaneously with a single sample. In contrast, analysis of skin biopsy biomarkers may not identify diseases compromising other organs aside from skin. Finally, skin biopsy requires the presence of a clinical lesion to determine the site of analysis and control skin. On the other hand, oral biofluids might reflect early changes that precede the development of a clinical skin lesion and, thus, might offer greater value for early diagnosis of the disease and real-time diagnostic values. Nevertheless, more studies are required to determine an adequate panel of biomarkers to use in saliva or GCF for psoriasis.

This article presents the classic limitation of a literature review because it does not follow a pre-established search and article selection design as in systematic reviews. In addition, we did not evaluate the quality of the included articles. However, this review allows us to answer the possible biomarkers in GCF and saliva to diagnose psoriasis.

## Figures and Tables

**Table 1:** GCF candidate biomarkers for Psoriasis.

Author, Year	Study Design	Population	Comparison (Control)	Technique	Outcomes	*p*
Mayer et al., 2013 [54]	Cross-sectional		Systemically healthy subjects (H), (*n* = 12).	ELISA	Most patients presented moderate/advance chronic periodontitis (79%).	
		Rheumatoid arthritis patients (RA), (*n* = 12)			Periodontal probing depths in the RA, PA and SSc groups were significantly worse than those of the H and RA+ groups.	**=0.0002**
		Rheumatoid arthritis patients undergoing anti-TNF-α therapy (RA+), (*n* = 10).			RA+ and H patients presented similar GCF levels of TNF-α (0.97 ± 0.52 and 1.07 ± 0.33 ng/site, respectively).	**=0.0001**
		Psoriatic arthritis patients (PA), (*n* = 12).			RA+ patients presented significantly lower GCF levels of TNF-α compared to RA, PA and SSc groups (0.97 ± 0.52, 1.07 ± 0.33, 1.42 ± 0.46, 1.97 ± 0.61, and 1.65 ± 0.57 ng/site, respectively)	**=0.0001**
		Systemic sclerosis patients (SSc), (*n* = 12).			No significant intergroup differences were reported between the GCF levels of TNF-α in RA, PA and SSc patients.	**=0.0001**
					Weak positive correlations were found between the GCF levels of TNF-α and the probing depth and gingival index in studied patients.	
Valenzuela et al., 2021 [25]	Cross-sectional	Moderate to severe Psoriasis subjects, (*n* = 42).	Systemically healthy subjects, (*n* = 39).	Multiplex bead-based immunoassay.	IL-18 GCF levels were significantly higher in psoriatic patients versus controls (mean, SD: 26.51 ± 10.46 pg/mL and 18.65 ± 5.17 pg/mL, respectively).	**<0.05**
					sE-selectin GCF levels were significantly lower in psoriasis patients versus healthy subjects (mean, SD: 31,490.35 ± 97,355.66 pg/mL and 201,873.5 ± 161,580.8 pg/mL, respectively).	**<0.05**
					No significant intergroup differences in the GCF levels of sICAM-1 were noticed.	>0.05
					Psoriasis influenced the levels of IL-18 and sE-selectin, whereas periodontitis influenced the levels of sICAM-1.	
					Diagnostic precision of IL-18 and sE-selectin for psoriasis based on ROC area were 0.77 and 0.68, respectively.	
Jimenez et al., 2021 [29]	Cross-sectional	Psoriatic subjects without periodontitis or mild periodontitis, (*n* = 11).	Systemically healthy subjects without periodontitis or mild periodontitis, (*n* = 21).	Multiplex bead-based immunoassay for IL-17A, IL-22, IL-23, S100A8 and S100A9	S100A8 GCF levels were overexpressed in psoriatic patients versus systemically healthy controls, regardless of periodontal status.	**<0.05**
					GCF levels of S100A8 correlated positively with psoriasis severity.	
		Psoriatic subjects with moderate or severe periodontitis, (*n* = 32).	Systemically healthy subjects with moderate or severe periodontitis, (*n* = 18).	ELISA for S100A7	IL-17A, IL-22. IL-23 and S100A7 showed no significant intergroup differences.	>0.05
					S100A9 exceeded the detection limits in all groups	

GCF, Gingival crevicular fluid; IL, Interleukin; pg, picograms; ng, nanograms; mL, milliliter. Bold, significant *p*-values.

## Data Availability

Not applicable.

## References

[1] Wu R., Zeng J., Yuan J., Deng X., Huang Y., Chen L., Zhang P., Feng H., Liu Z., Wang Z. (2018). MicroRNA-210 overexpression promotes psoriasis-like inflammation by inducing Th1 and Th17 cell differentiation. J. Clin. Investig..

[2] Meneguin S., de Godoy N.A., Pollo C.F., Miot H.A., de Oliveira C. (2020). Quality of life of patients living with psoriasis: A qualitative study. BMC Dermatol..

[3] Parisi R., Iskandar I.Y.K., Kontopantelis E., Augustin M., Griffiths C.E.M., Ashcroft D.M., Global Psoriasis A. (2020). National, regional, and worldwide epidemiology of psoriasis: Systematic analysis and modelling study. BMJ.

[4] Odorici G., Paganelli A., Peccerillo F., Serra J., Chester J., Kaleci S., Pellacani G., Conti A. (2021). Moderate to severe psoriasis: A single-center analysis of gender prevalence. Ital. J. Dermatol. Venerol..

[5] Choi B.G., Hong J.Y., Hong J.R., Hur M.S., Kim S.M., Lee Y.W., Choe Y.B., Ahn K.J. (2019). The IL17F His161Arg polymorphism, a potential risk locus for psoriasis, increases serum levels of interleukin-17F in an Asian population. Sci. Rep..

[6] Romani J., Julia M., Lozano F., Munoz-Santos C., Guilabert A., Carrascosa J.M., Rigla M., Luelmo J. (2015). Toll-like receptor 9 promoter polymorphism as a predictive factor of narrow-band UVB phototherapy response in patients with psoriasis. Photodermatol. Photoimmunol. Photomed..

[7] Shi H.J., Zhou H., Ma A.L., Wang L., Gao Q., Zhang N., Song H.B., Bo K.P., Ma W. (2019). Oxymatrine therapy inhibited epidermal cell proliferation and apoptosis in severe plaque psoriasis. Br. J. Dermatol..

[8] Aragona E., Rania L., Postorino E.I., Interdonato A., Giuffrida R., Cannavo S.P., Puzzolo D., Aragona P. (2018). Tear film and ocular surface assessment in psoriasis. Br. J. Ophthalmol..

[9] Zargari O., Leyli E.K., Azimi S.Z. (2018). Nail Involvement in Patients with Psoriatic Arthritis in Northern Iran. Autoimmune Dis..

[10] Ganzetti G., Campanati A., Santarelli A., Pozzi V., Molinelli E., Minnetti I., Brisigotti V., Procaccini M., Emanuelli M., Offidani A. (2014). Periodontal disease: An oral manifestation of psoriasis or an occasional finding?. Drug Dev. Res..

[11] Catapano M., Vergnano M., Romano M., Mahil S.K., Choon S.E., Burden A.D., Young H.S., Carr I.M., Lachmann H.J., Lombardi G. (2020). IL-36 Promotes Systemic IFN-I Responses in Severe Forms of Psoriasis. J. Investig. Dermatol..

[12] Sobhan M.R., Farshchian M., Hoseinzadeh A., Ghasemibasir H.R., Solgi G. (2016). Serum Levels of IL-10 and IL-22 Cytokines in Patients with Psoriasis. Iran. J. Immunol..

[13] Oliveira A.N., Simoes M.M., Simoes R., Malachias M.V.B., Rezende B.A. (2019). Cardiovascular Risk in Psoriasis Patients: Clinical, Functional and Morphological Parameters. Arq. Bras. Cardiol..

[14] Schlaak J.F., Buslau M., Jochum W., Hermann E., Girndt M., Gallati H., Meyer zum Buschenfelde K.H., Fleischer B. (1994). T cells involved in psoriasis vulgaris belong to the Th1 subset. J. Investig. Dermatol..

[15] Boyman O., Hefti H.P., Conrad C., Nickoloff B.J., Suter M., Nestle F.O. (2004). Spontaneous development of psoriasis in a new animal model shows an essential role for resident T cells and tumor necrosis factor-alpha. J. Exp. Med..

[16] Austin L.M., Ozawa M., Kikuchi T., Walters I.B., Krueger J.G. (1999). The majority of epidermal T cells in Psoriasis vulgaris lesions can produce type 1 cytokines, interferon-gamma, interleukin-2, and tumor necrosis factor-alpha, defining TC1 (cytotoxic T lymphocyte) and TH1 effector populations: A type 1 differentiation bias is also measured in circulating blood T cells in psoriatic patients. J. Investig. Dermatol..

[17] Infante-Duarte C., Horton H.F., Byrne M.C., Kamradt T. (2000). Microbial lipopeptides induce the production of IL-17 in Th cells. J. Immunol..

[18] Kimura A., Naka T., Kishimoto T. (2007). IL-6-dependent and -independent pathways in the development of interleukin 17-producing T helper cells. Proc. Natl. Acad. Sci. USA.

[19] Arnold I.C., Mathisen S., Schulthess J., Danne C., Hegazy A.N., Powrie F. (2016). CD11c(+) monocyte/macrophages promote chronic Helicobacter hepaticus-induced intestinal inflammation through the production of IL-23. Mucosal Immunol..

[20] Wohn C., Ober-Blobaum J.L., Haak S., Pantelyushin S., Cheong C., Zahner S.P., Onderwater S., Kant M., Weighardt H., Holzmann B. (2013). Langerin(neg) conventional dendritic cells produce IL-23 to drive psoriatic plaque formation in mice. Proc. Natl. Acad. Sci. USA.

[21] Krueger J.G., Wharton K.A., Schlitt T., Suprun M., Torene R.I., Jiang X., Wang C.Q., Fuentes-Duculan J., Hartmann N., Peters T. (2019). IL-17A inhibition by secukinumab induces early clinical, histopathologic, and molecular resolution of psoriasis. J. Allergy Clin. Immunol..

[22] Kim W.B., Jerome D., Yeung J. (2017). Diagnosis and management of psoriasis. Can. Fam. Physician.

[23] Gisondi P., Bellinato F., Girolomoni G. (2020). Topographic Differential Diagnosis of Chronic Plaque Psoriasis: Challenges and Tricks. J. Clin. Med..

[24] De Rosa G., Mignogna C. (2007). The histopathology of psoriasis. Reumatismo.

[25] Valenzuela F., Fernandez J., Jimenez C., Cavagnola D., Mancilla J.F., Astorga J., Hernandez M., Fernandez A. (2021). Identification of IL-18 and Soluble Cell Adhesion Molecules in the Gingival Crevicular Fluid as Novel Biomarkers of Psoriasis. Life.

[26] Cardoso P.R., Lima E.V., Lima M.M., Rego M.J., Marques C.D., Pitta Ida R., Duarte A.L., Pitta M.G. (2016). Clinical and cytokine profile evaluation in Northeast Brazilian psoriasis plaque-type patients. Eur. Cytokine Netw..

[27] Ganzetti G., Campanati A., Santarelli A., Sartini D., Molinelli E., Brisigotti V., Di Ruscio G., Bobyr I., Emanuelli M., Offidani A. (2016). Salivary interleukin-1beta: Oral inflammatory biomarker in patients with psoriasis. J. Int. Med. Res..

[28] Califf R.M. (2018). Biomarker definitions and their applications. Exp. Biol. Med..

[29] Jimenez C., Carvajal D., Hernandez M., Valenzuela F., Astorga J., Fernandez A. (2021). Levels of the interleukins 17A, 22, and 23 and the S100 protein family in the gingival crevicular fluid of psoriatic patients with or without periodontitis. An. Bras. Dermatol..

[30] Chandrasekaran A.R., MacIsaac M., Vilcapoma J., Hansen C.H., Yang D., Wong W.P., Halvorsen K. (2021). DNA Nanoswitch Barcodes for Multiplexed Biomarker Profiling. Nano Lett..

[31] Papagerakis P., Zheng L., Kim D., Said R., Ehlert A.A., Chung K.K.M., Papagerakis S. (2019). Saliva and Gingival Crevicular Fluid (GCF) Collection for Biomarker Screening. Methods Mol. Biol..

[32] Fatima T., Khurshid Z., Rehman A., Imran E., Srivastava K.C., Shrivastava D. (2021). Gingival Crevicular Fluid (GCF): A Diagnostic Tool for the Detection of Periodontal Health and Diseases. Molecules.

[33] Guentsch A., Kramesberger M., Sroka A., Pfister W., Potempa J., Eick S. (2011). Comparison of gingival crevicular fluid sampling methods in patients with severe chronic periodontitis. J. Periodontol..

[34] Goodson J.M. (2003). Gingival crevice fluid flow. Periodontology 2000.

[35] Shiloah J., Hovious L.A. (1993). The role of subgingival irrigations in the treatment of periodontitis. J. Periodontol..

[36] Greenstein G., Berman C., Jaffin R. (1986). Chlorhexidine. An adjunct to periodontal therapy. J. Periodontol..

[37] Kirtiloglu T., Keskiner I., Sahin M., Kirtiloglu B., Aygul S., Sakallioglu U., Acikgoz G. (2020). Assessment of the half-life of cationic periodontal pocket irrigation. BMC Oral Health.

[38] Sahrmann P., Sener B., Ronay V., Attin T., Schmidlin P.R. (2012). Clearance of topically-applied PVP-iodine as a solution or gel in periodontal pockets in men. Acta Odontol. Scand..

[39] Griffiths G.S., Wilton J.M., Curtis M.A. (1992). Contamination of human gingival crevicular fluid by plaque and saliva. Arch. Oral Biol..

[40] Challacombe S.J., Russell M.W., Hawkes J.E., Bergmeier L.A., Lehner T. (1978). Passage of immunoglobulins from plasma to the oral cavity in rhesus monkeys. Immunology.

[41] Khurshid Z., Mali M., Naseem M., Najeeb S., Zafar M.S. (2017). Human Gingival Crevicular Fluids (GCF) Proteomics: An Overview. Dent. J..

[42] Wassall R.R., Preshaw P.M. (2016). Clinical and technical considerations in the analysis of gingival crevicular fluid. Periodontology 2000.

[43] Bostanci N., Belibasakis G.N. (2018). Gingival crevicular fluid and its immune mediators in the proteomic era. Periodontology 2000.

[44] Loe H., Holm-Pedersen P. (1965). Absence and Presence of Fluid from Normal and Inflamed Gingivae. Periodontics.

[45] Ghallab N.A. (2018). Diagnostic potential and future directions of biomarkers in gingival crevicular fluid and saliva of periodontal diseases: Review of the current evidence. Arch. Oral Biol..

[46] Oswal S., Dwarakanath C.D. (2010). Relevance of gingival crevice fluid components in assessment of periodontal disease—A critical analysis. J. Indian Soc. Periodontol..

[47] Gupta S., Chhina S., Arora S.A. (2018). A systematic review of biomarkers of gingival crevicular fluid: Their predictive role in diagnosis of periodontal disease status. J. Oral Biol. Craniofac. Res..

[48] Arvikar S.L., Hasturk H., Strle K., Stephens D., Bolster M.B., Collier D.S., Kantarci A., Steere A.C. (2021). Periodontal inflammation and distinct inflammatory profiles in saliva and gingival crevicular fluid compared with serum and joints in rheumatoid arthritis patients. J. Periodontol..

[49] Prieto D., Gonzalez C., Weber L., Realini O., Pino-Lagos K., Bendek M.J., Retamal I., Beltran V., Riedemann J.P., Espinoza F. (2021). Soluble neuropilin-1 in gingival crevicular fluid is associated with rheumatoid arthritis: An exploratory case-control study. J. Oral Biol. Craniofac. Res..

[50] Ma X., Wang Y., Wu H., Li F., Feng X., Xie Y., Xie D., Wang W., Lo E.C.M., Lu H. (2021). Periodontal health related-inflammatory and metabolic profiles of patients with end-stage renal disease: Potential strategy for predictive, preventive, and personalized medicine. EPMA J..

[51] Salvi G.E., Yalda B., Collins J.G., Jones B.H., Smith F.W., Arnold R.R., Offenbacher S. (1997). Inflammatory mediator response as a potential risk marker for periodontal diseases in insulin-dependent diabetes mellitus patients. J. Periodontol..

[52] Sakai A., Ohshima M., Sugano N., Otsuka K., Ito K. (2006). Profiling the cytokines in gingival crevicular fluid using a cytokine antibody array. J. Periodontol..

[53] Valenzuela F., Fernandez J., Aroca M., Jimenez C., Albers D., Hernandez M., Fernandez A. (2020). Gingival Crevicular Fluid Zinc- and Aspartyl-Binding Protease Profile of Individuals with Moderate/Severe Atopic Dermatitis. Biomolecules.

[54] Mayer Y., Elimelech R., Balbir-Gurman A., Braun-Moscovici Y., Machtei E.E. (2013). Periodontal condition of patients with autoimmune diseases and the effect of anti-tumor necrosis factor-alpha therapy. J. Periodontol..

[55] Forouzandeh M., Besen J., Keane R.W., de Rivero Vaccari J.P. (2020). The Inflammasome Signaling Proteins ASC and IL-18 as Biomarkers of Psoriasis. Front. Pharmacol..

[56] Zhang F., Liu E., Radaic A., Yu X., Yang S., Yu C., Xiao S., Ye C. (2021). Diagnostic potential and future directions of matrix metalloproteinases as biomarkers in gingival crevicular fluid of oral and systemic diseases. Int. J. Biol. Macromol..

[57] Kelly M., Vardhanabhuti B., Luck P., Drake M.A., Osborne J., Foegeding E.A. (2010). Role of protein concentration and protein-saliva interactions in the astringency of whey proteins at low pH. J. Dairy Sci..

[58] Golatowski C., Salazar M.G., Dhople V.M., Hammer E., Kocher T., Jehmlich N., Volker U. (2013). Comparative evaluation of saliva collection methods for proteome analysis. Clin. Chim. Acta.

[59] Kluknavska J., Krajcikova K., Bolerazska B., Maslankova J., Ohlasova J., Timkova S., Drotarova Z., Vaskova J. (2021). Possible prognostic biomarkers of periodontitis in saliva. Eur. Rev. Med. Pharmacol. Sci..

[60] Klimiuk A., Maciejczyk M., Choromanska M., Fejfer K., Waszkiewicz N., Zalewska A. (2019). Salivary Redox Biomarkers in Different Stages of Dementia Severity. J. Clin. Med..

[61] Brandtzaeg P. (2007). Do salivary antibodies reliably reflect both mucosal and systemic immunity?. Ann. N. Y. Acad. Sci..

[62] Choromanska M., Klimiuk A., Kostecka-Sochon P., Wilczynska K., Kwiatkowski M., Okuniewska N., Waszkiewicz N., Zalewska A., Maciejczyk M. (2017). Antioxidant Defence, Oxidative Stress and Oxidative Damage in Saliva, Plasma and Erythrocytes of Dementia Patients. Can Salivary AGE be a Marker of Dementia?. Int. J. Mol. Sci..

[63] Yoshizawa J.M., Schafer C.A., Schafer J.J., Farrell J.J., Paster B.J., Wong D.T. (2013). Salivary biomarkers: Toward future clinical and diagnostic utilities. Clin. Microbiol. Rev..

[64] Skutnik-Radziszewska A., Maciejczyk M., Flisiak I., Kolodziej J.K.U., Kotowska-Rodziewicz A., Klimiuk A., Zalewska A. (2020). Enhanced Inflammation and Nitrosative Stress in the Saliva and Plasma of Patients with Plaque Psoriasis. J. Clin. Med..

[65] Liu R., Yang Y., Yan X., Zhang K. (2013). Abnormalities in cytokine secretion from mesenchymal stem cells in psoriatic skin lesions. Eur. J. Dermatol..

[66] Mastrolonardo M., Alicino D., Zefferino R., Pasquini P., Picardi A. (2007). Effect of psychological stress on salivary interleukin-1beta in psoriasis. Arch. Med. Res..

[67] Bottoni U., Tiriolo R., Pullano S.A., Dastoli S., Amoruso G.F., Nistico S.P., Fiorillo A.S. (2016). Infrared Saliva Analysis of Psoriatic and Diabetic Patients: Similarities in Protein Components. IEEE Trans. Biomed. Eng..

[68] del Castillo Carrillo L.F., Schwarz W., Hornstein O.P. (1981). Immunoglobulins in serum, whole saliva, and parotid saliva of male healthy and psoriatic individuals. Arch. Dermatol. Res..

[69] Koh D., Yang Y., Khoo L., Nyunt S.Z., Ng V., Goh C.L. (2004). Salivary immunoglobulin A and lysozyme in patients with psoriasis. Ann. Acad. Med. Singap..

[70] Syrjanen S.M. (1983). Chemical analysis of parotid saliva and lacrimal fluid in psoriatics. Arch. Dermatol. Res..

[71] Belstrom D., Eiberg J.M., Enevold C., Grande M.A., Jensen C.A.J., Skov L., Hansen P.R. (2020). Salivary microbiota and inflammation-related proteins in patients with psoriasis. Oral Dis..

[72] Soudan R.A., Daoud S.A., Mashlah A.M. (2011). Study of some salivary changes in cutaneous psoriatic patients. Saudi Med. J..

